# Surgery

**Published:** 1913-09

**Authors:** A. Rendle Short


### SURGERY.

**Tetanus.**—In spite of the discouraging results of treatment in this disease, efforts are being made in a variety of directions to advance our knowledge and improve our methods of dealing with it.

*Pathology.*—It is well recognised now that the toxin of tetanus reaches the central nervous system by way of the nerves, not by the blood-stream. Interesting confirmation of this is afforded by the phenomenon of the so-called "cephalic type." Wounds of the seventh nerve area which give rise to tetanus are accompanied by facial paralysis from strangulation of the swollen nerve in the aqueduct of Fallopius.

The causation of post-operative tetanus has been the subject of an interesting paper by Richardson,[1] who concludes that it arises in particular districts, especially in Northumberland, and under circumstances where the catgut and other surgical appliances could not possibly have been the agents conveying the infection. He believes that the symptoms are not due to tetanus bacilli, but to an intestinal infection with a similar organism which causes the "louping ill" in sheep, and which lies latent until it is brought into activity by the general upset of the operation. Such a theory must be very solacing to the unfortunate surgeon, but the explanation will not, of course, cover cases in which there are several successive cases.

[1] Richardson, *Brit. M. J.*, 1909, i. 948.

In the tropics, where intramuscular injections of quinine are often given for malaria, it has often been observed that tetanus has followed, in spite of great care in boiling the needle and solution. Investigation shows that a few tetanus bacilli may lurk harmlessly in an aseptic wound in an animal, which when an injection of quinine is given, even in a remote region, light up the disease in an active or even fatal form. Probably in the human cases after quinine there were already a few quiescent tetanus bacilli which had entered some forgotton abrasion, and which in some mysterious way were now activated. It is suggested here also that the bowel may be the lurking place, and in the tropics bacilli like tetanus often occur in the stools.[1]

*Prevention of Tetanus.*—Although a thorough cleansing of the earth-infected wound when first seen probably helps to reduce the danger, yet it is very far from being a perfect protection. In several cases at the Bristol Royal Infirmary the wound was efficiently dealt with only a few hours after infliction, and nevertheless tetanus supervened. In America, therefore, doses of anti-tetanic serum are given to patients with an earth-infected wound as a routine, and several long series are reported in which no tetanus ever developed, but it is difficult to say that any of them would have manifested the disease apart from the prophylactic treatment. On the other hand, Jacobson and Pease[2] mention nineteen cases in the literature (to which we can add another) in which tetanus came on in spite of the serum having been given. The great majority of them recovered, and the incubation period was prolonged. Probably, therefore, there is real benefit. In experimental animals anti-tetanic serum is far more successful as a prophylactic than as a cure.

*Treatment.*—We shall not here refer to the obvious treatment, such as rest and seclusion, feeding, sedatives, cleansing of the wound, etc. A goodly number of cases would recover with but the simplest treatment. There is always a fair prospect of survival if the incubation period is over about sixteen days. Wounds of the leg are more favourable than those of other parts, because the toxins have to travel up a greater length of nerve.

Numerous attempts have been made to obtain better results with the antitoxin, but all statistics are much vitiated by two fallacies—first, that of picking out reports (usually of recovery) from the literature instead of quoting consecutive series; second, that of making no distinction between severe cases with a short incubation period and the milder ones which would recover anyhow.

Colossal doses (which are very expensive) are said to

[1] Semple (Review in), *Brit. M. J.*, 1911, ii. 390.

[2] Jacobson and Pease, *Ann. Surg.*, 1906, xliv. 321.

have cured 15 out of 20 cases (Jacobson and Pease). In each of these 300 to 500 c.c. were injected. It is by no means clear that they were severe cases.

*Intracerebral injection* is or ought to be abandoned. Of 88 collected cases (*v.* Graff) 80 per cent. died ; Sawamura reports another series with a 67 per cent. mortality. In several cases abscess of the brain has supervened.

*Intraneural injection* is probably more reliable than subcutaneous or intravenous, and gives much better results in rabbits (Sawamura).

*Intraspinal injection* is well spoken of, but definite evidence is not very conclusive. Rogers saved 4 cases out of 7 by giving the antitoxin by all four routes—intraneural, intraspinal, intravenous, and subcutaneous—to each patient. Hofmann[1] reports 30 consecutive cases. In the first 13 only subcutaneous injections were given, and 7 died (=53.8 per cent.) ; in the last 16, treated by intraspinal injection, only 2 died, of these 4 were very severely affected. Ashhurst[2] treated 5 patients within twelve hours of the onset of symptoms by huge doses (3,000 c.c.) of antitoxin given by all routes together, and only one died ; he also saved 4 out of 11 cases in which the incubation period was less than ten days, but only one appears to have had severe symptoms. Altogether he saved 44 per cent. of 23 cases, which is fairly good. At the Bristol Royal Infirmary, 9 out of 31 have recovered. Jacobson and Pease, quoting from two hundred American hospital cases, say that of acute cases 17 per cent. recover, and of chronic cases about 56 per cent.

*Baccelli's Carbolic Treatment.*—On paper the results obtained by this method infinitely surpass those of every other, but the statistics are compiled in a way that inspires distrust. Baccelli[3] reports that 92 out of 94 " severe " cases recovered, and also 16 out of 38 " very severe " cases. Apparently there were no mild cases treated ! The successes are isolated records of individual cures picked out of Italian journals, scores of authors contributing a case or two each.

The treatment is to inject a 3 per cent. solution of carbolic acid hypodermically, rising from 3 grain doses until 30 to 45 grains are given in twenty-four hours. Carboluria results, but is to be neglected.

*Magnesium Sulphate Treatment.*—The injection of 2 or 3 c.c. of 25 per cent. solution of $MgSO_4$ into the spinal theca is a modern treatment of tetanus which has become rather popular. It is often successful in animals in quieting the spasms, but results are conflicting as to whether it saves lives. In the

[1] Hofmann, *Beitr. zklin. Chir.*, 1907, lv. 697.

[2] Ashhurst, *Am. J. M. Sc.*, 1913, cxlvi. 77.

[3] Baccelli, *Berl. klin. Wchnschr.*, 1911, xlviii. 1021.

human subject the evidence is at present scanty. Phillips[1] reports a consecutive series of 7 patients in which 4 recovered. Out of 28 cases reported in the literature by various authors 16 recovered. The method is by no means free from risk—one patient died and another was only saved by artificial respiration. It would be wise to keep the patient's head up.

*Other Methods.*—In Russia good results are reported by Krokiewicz,[2] who saved 13 out of 16 patients by giving injections of 10 grains of sheep's brain in saline, the idea being to take advantage of the affinity of the toxin for nervous matter so as to fix it harmlessly.

*Summary.*—It is probably well to give antitoxin both subcutaneously and by intraspinal injection, and if the symptoms persist to try the magnesium sulphate treatment. When circumstances do not favour a lumbar puncture, the very simple carbolic acid treatment is certainly worth a serious trial.

A. Rendle Short.

### OPHTHALMOLOGY.

[1] Phillips, *Proc. Roy. Soc. Med.*, 1910, ii. (Med. section), 39.

[2] Keen, *Surgery*, article "Tetanus."

